# Chromosome 15q11-q13 copy number gain detected by array-CGH in two cases with a maternal methylation pattern

**DOI:** 10.1186/1755-8166-7-32

**Published:** 2014-05-16

**Authors:** Ee-Shien Tan, Min-Hwee Yong, Eileen CP Lim, Zhi-hui Li, Maggie SY Brett, Ene-Choo Tan

**Affiliations:** 1 Genetics Service, KK Women’s & Children’s Hospital, 100 Bukit Timah Road 229899 Singapore, Singapore; 2 Cytogenetics Laboratory, KK Women’s & Children’s Hospital, 100 Bukit Timah Road 229899 Singapore, Singapore; 3 KK Research Laboratory, KK Women’s & Children’s Hospital, 100 Bukit Timah Road 229899 Singapore, Singapore; 4 Genomax Technologies Pte Ltd, 51 Science Park Road, #04-15 117586 Singapore, Singapore; 5 Office of Clinical Sciences, Duke-NUS Graduate Medical School, 8 College Road 169857 Singapore, Singapore

**Keywords:** 15q duplication syndrome, Array comparative genomic hybridization (aCGH), Copy number gain, Autism, Developmental delay, Fluorescence in situ hybridization (FISH), Marker chromosome

## Abstract

**Background:**

The 15q11-q13 region contains many low copy repeats and is well known for its genomic instability. Several syndromes are associated with genomic imbalance or copy-number-neutral uniparental disomy. We report on two patients: Patient 1 is a boy with developmental delay and autism; and Patient 2 is a girl with developmental delay, hypotonia and dysmorphism. We performed analyses to delineate their dosage in the 15q region, determine whether the patients’ dosage correlates with phenotypic severity, and whether genes in the amplified regions are significantly associated with identified functional networks.

**Results:**

For the proximal region of 15q, molecular cytogenetic analysis with Agilent oligonucleotide array showed a copy number of 3 for Patient 1 and a copy number of 4 for Patient 2. Fluorescent in situ hybridization analysis of Patient 2 showed two different populations of cells with different marker chromosomes. Methylation analysis of the amplified region showed that the extra copies of small nuclear ribonucleoprotein polypeptide N gene were of maternal origin. Phenotypic severity did not correlate with the size and dosage of 15q, or whether the amplification is interstitial or in the form of a supernumerary marker. Pathway analysis showed that in Patient 2, the main functional networks that are affected by the genes from the duplicated/triplicated regions are developmental disorder, neurological disease and hereditary disease.

**Conclusions:**

The 15q11-q13 gains that were found in both patients could explain their phenotypic presentations. This report expands the cohort of patients for which 15q11-q13 duplications are molecularly characterized.

## Background

The 15q11-q13 region is a hotspot for recombination. Several breakpoint (BP) regions have clusters that contain low copy repeats and segmental duplications [1]. Chiasmata frequency in the region is known to be higher than in other chromosomal regions [2]. As a consequence, the region is prone to having deletions, duplications and rearrangements. In addition to frequent genomic rearrangements, this chromosomal region is also highly regulated by methylation, and the allele that is expressed for specific genes is based on the parental origin of the chromosome. The presence of imprinted genes in this region means that there may be phenotypic consequences even if the rearrangements are copy number neutral and results in no genomic imbalance.

Genomic disorders mapped to this region include Angelman syndrome (Online Mendelian Inheritance in Man (OMIM) #105830) and Prader-Willi syndrome (OMIM #176270). In the majority of cases, both of the syndromes are due to either deletion or uniparental disomy. Of the remaining cases of Angelman syndrome, approximately 10-15% are due to *UBE3A* mutations and 2-4% are due to imprinting centre defect. Less than 1% of the remaining Prader-Willi syndrome cases are due to imprinting centre defect [3]. Cytogenetically visible duplications (OMIM #608636) and marker chromosomes from derivatives of this region are also common. The high frequency of such cases gave rise to a clinically recognizable disorder called 15q duplication syndrome, with some common neurobehavioural phenotypes [4]. Copy numbers of three, four, five and six have all been reported for this region [5-7].

Most copy number gains in this region are due to translocations, inversions and supernumerary marker chromosomes (sSMC). Interstitial duplications/triplications and balanced translocations (which do not result in copy number changes) are more infrequent [8]. To add to the clinical phenotype of 15q duplication cases, we present two patients - a Malay boy with partial trisomy 15q and a Chinese girl with mosaic partial tetrasomy 15q. We conducted analyses to delineate their amplification in the 15q region, analyze the region at the gene level, and determine whether genes within the amplified regions are significantly associated with identified functional networks.

### Presentation of cases

The two patients described in this study were participants in a study to identify genomic imbalance in patients with developmental delay/multiple congenital anomalies. They were recruited from the genetics outpatient clinics of the KK Women’s and Children’s Hospital, Singapore. The study was approved by the SingHealth Institutional Review Board, which oversees all research studies in the hospital. The patients were recruited with the written informed consent of their parents.

Patient 1 is the 6^th^ child of healthy, unrelated parents of Malay ancestry. His mother was 33 years old at the time of his birth. He has an older brother with autism spectrum disorder, but four other siblings are phenotypically normal. He was delivered at full term with a birth weight of 2950 g and there were no perinatal issues. He first presented at 5 years and 8 months of age with severe language delay, hyperactivity and a preoccupation with water. His verbal language was limited to the repetition of a few words and pointing for needs. His teachers reported that it was difficult to engage him in his pre-school activities. He was diagnosed with autism spectrum disorder and intellectual impairment. Clinical examination revealed a well thrived child with no dysmorphic features. Fragile X testing result was normal.

The second patient was a girl of Chinese descent born at 39 weeks gestation with a birth weight of 3234 g, length of 45.5 cm and head circumference of 32 cm. Her Apgar scores at 1 and 5 minutes were both 9. She is the third child of a non-consanguineous marriage with no family history of autism or learning impairment. Her mother was 28 years old and her father 29 years old at the time of her birth. She was first noted to have gross motor delay at 9 months of age. The initial investigations, including a thyroid function test and metabolic screen, were normal. On further review, she continued to have significant developmental delay. At two years of age, she was able to walk with support and she spoke no clear words. Assessment using the Age and States Questionnaire, Third Edition, showed normal scores for fine motor and personal-social skills, and a borderline score in communication skills. The patient had significant delays in gross motor and problem-solving skills.

On physical examination, her height and weight were at the 90^th^ percentile and her head circumference was at the 97^th^ percentile. She had truncal hypotonia and pigmentation, irregular pigmentation on her lower limbs, protruding tongue and hypertelorism, but no other dysmorphic features. Chromosome culture revealed an abnormal female karyotype, with two cell lines that showed a different additional marker chromosome in each cell line.

## Results

### Karyotype analysis

**Figure 1:**
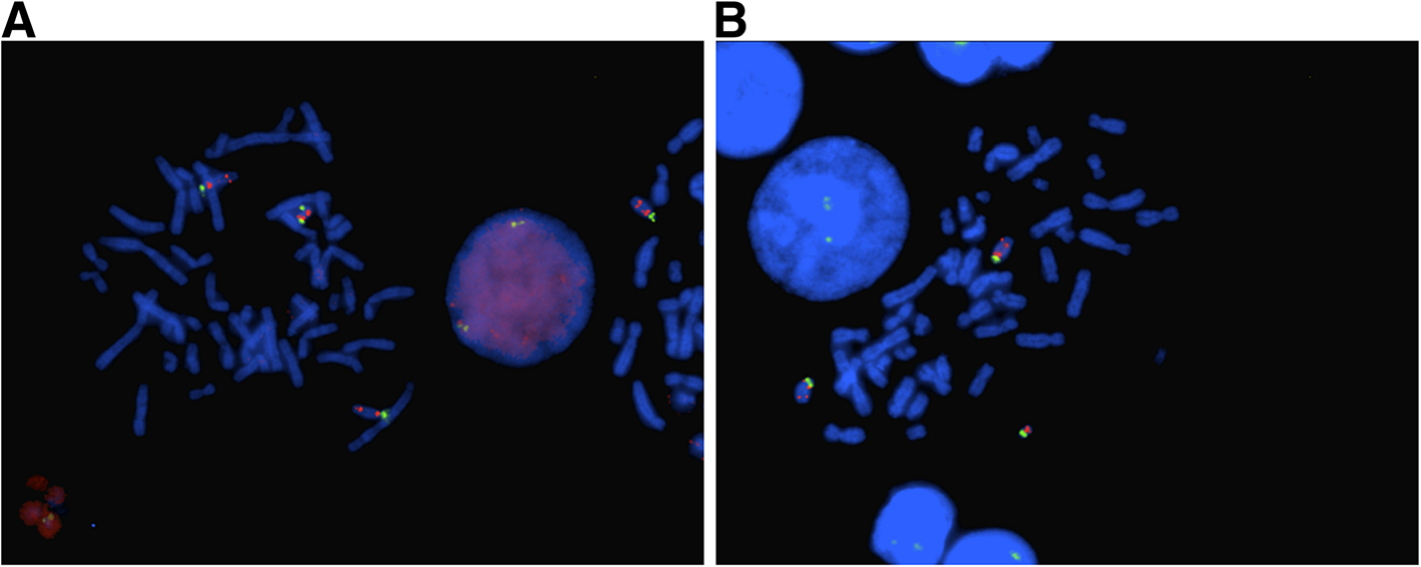
**FISH analysis of patient 2. (A)** 2 green and 2 red hybridization signals were seen on the larger marker corresponding to centromeric 15 (D15Z1) and *SNRPN* respectively **(B)** 1 green and 1 red hybridization signals were seen on the smaller marker corresponding to centromeric 15 (D15Z1) and *SNRPN* respectively (Abbreviations: FISH: Fluorescent in situ hybridization, *SNRPN*: Small nuclear ribonucleoprotein polypeptide N).

Karyotype information on Patient 1 was unavailable. For Patient 2, karyotype is reported as 47,XX,+mar.ish del(15)(q12)(SNRPN+,D15Z1+)[14]/47,XX,+mar.ish psu dic(15;15)(SNRPN++,D15Z1++) [4]. Two different markers were identified. The larger marker chromosome was found in 14 out of 18 metaphases analyzed. Fluorescent in situ hybridization (FISH) analysis showed two centromeric 15 signals (D15Z1) and two signals localized to *SNRPN* on this marker chromosome (Figure 1A). A smaller marker chromosome was found in the other 4 metaphases. FISH analysis on this smaller marker chromosome showed one centromeric 15 signal and one signal localized to *SNRPN* (Figure 1B). Hence, cytogenetic and FISH analysis showed that this patient is mosaic for the two marker chromosomes, with 14 out of 18 cells harboring the marker chromosome with two copies of proximal 15q and the remaining 4 cells harboring the marker chromosome with one copy of proximal 15q, corresponding to partial tetrasomy and partial trisomy 15q.

### Array-based copy number analysis

**Figure 2:**
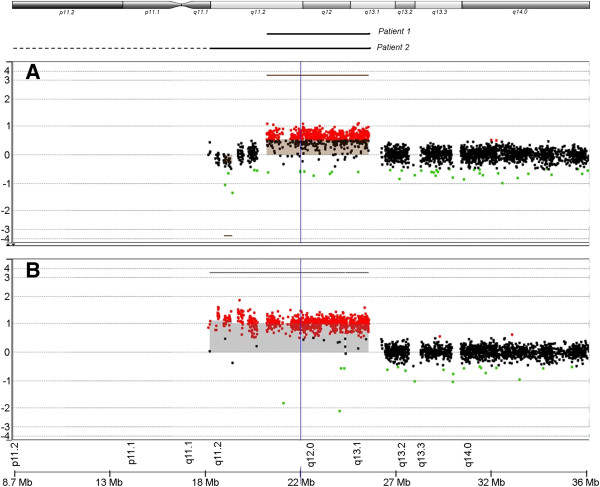
**Screenshot of analysis with Genomic Workbench Lite for Array-CGH results.** Chart shows the gain in copy number from 15q11 to 15q13 for **(A)** Patient 1 and **(B)** Patient 2. The regions with the copy number gain corresponding to the CGH results were indicated by the solid lines below the ideogram. (*Abbreviations:* CGH; Comparative genomic hybridization; Mb: million base-pairs).

For Patient 1, analysis showed a gain in copy number from 21,213,950 to 26,208,646, involving 710 probes with a mean log_2_ ratio of 0.5585 for the proximal region of the long arm of chromosome 15 (Figure 2A). For Patient 2, the gain was from 18,362,555 to 26,208,646, involving 865 probes with mean log_2_ ratio of 1.0239 (Figure 2B). From the position of the probes, the estimated minimum size of the gain for Patient 1 was 4,994,696 basepairs (bp) while the maximum size was 6,045,492 bp. For Patient 2, the minimum size for the gain was 7,846,091 bp. The maximum size could not be determined as the gain started from the first probe for the chromosome. The list of known genes in the amplified region is shown in Table 1.

**Table 1:** List of genes for the duplicated region for patient 2

**Symbol**	**Gene name**	**HI(D)**^**1**^	**HI(I)**^**2**^	**TS(I)**^**3**^	**Imprinting**^**4**^
*POTEB*	POTE ankyrin domain family, member B	-	-	-	-
*OR4M2*	Olfactory receptor, family 4, subfamily M, member 2	93.7	-	-	-
*OR4N4*	Olfactory receptor, family 4, subfamily N, member 4	78.3	-	-	-
*GOLGA6L1*	Golgin A6 family-like 1	-	-	-	-
*TUBGCP5*	Tubulin-gamma complex-associated protein 5	-	-	-	-
*CYFIP1*	Cytoplasmic FMRP interacting protein 1	53.3	-	-	-
*NIPA2*	Not imprinted in Prader-Willi syndrome/Angelman syndrome 2	52.9	0	0	-
NIPA1*	Not imprinted in Prader-Willi syndrome/Angelman syndrome 1	25.3	0	0	-
*GOLGA6L2*	Golgin A6 family-like 2	-	-	-	-
*MKRN3*	Makorin ring finger protein 3	98.7	0	0	P
*MAGEL2*	MAGE-like 2	-	-	-	P
NDN*	Necdin, melanoma antigen (MAGE) family member	36.2	-	-	P
NPAP1*	Nuclear pore associated protein 1	93.6	-	-	-
SNRPN*	Small nuclear ribonucleoprotein polypeptide N	11.3	-	-	P
*SNURF*	SNRPN upstream reading frame	64.6	-	-	P
UBE3A*^	Ubiquitin protein ligase E3A	23.2	3	0	M
ATP10A*	ATPase, class V, type 10A	74.0	0	0	M
GABRB3*	Gamma-aminobutyric acid (GABA) A receptor, beta-3	5.2	0	0	-
*GABRA5*	Gamma-aminobutyric acid (GABA) A receptor, alpha-5	25.5	-	-	-
*GABRG3*	Gamma-aminobutyric acid (GABA) A receptor, gamma-3	83.8	-	-	-
OCA2*	Pink-eye dilution, murine, homolog of (oculocutaneous albinism II)	71.5	0	0	-
HERC2*	HECT domain and RCC1-like domain 2	42.7	-	-	-

Notes: Gene list and names according to Decipher (https://decipher.sanger.ac.uk/). The list for Patient #1 is from *MKRN3* to *HERC2*.

*OMIM morbid genes.

^Associated with developmental disorders according to the Developmental Disorders Genotype-Phenotype Database (DDG2P).

^1^HI(D) Haploinsufficiency scores from Decipher: values from 0-100%. Low values (0-10%) indicate more likely to exhibit Haploinsufficiency.

^2^HI(I) ISCA Haploinsufficiency Score (0 = no evidence available, 1 = Little evidence, 2 = Some evidence, 3 = Sufficient evidence for dosage pathogenicity).

^3^TS(I) ISCA Triplosensitivity Score (0 = no evidence available, 1 = Little evidence, 2 = Some evidence, 3 = Sufficient evidence for dosage pathogenicity) (http://www.ncbi.nlm.nih.gov/projects/dbvar/ISCA/).

^4^For last column: M = expressed from maternal allele, P = expressed from paternal allele.

*Abbreviations:* FMRP; Fragile X mental retardation protein; HECT: Homologous to the E6-AP carboxyl terminus; SNRPN; small nuclear ribonucleoprotein polypeptide N.

### Quantitative polymerase chain reaction analysis

**Figure 3:**
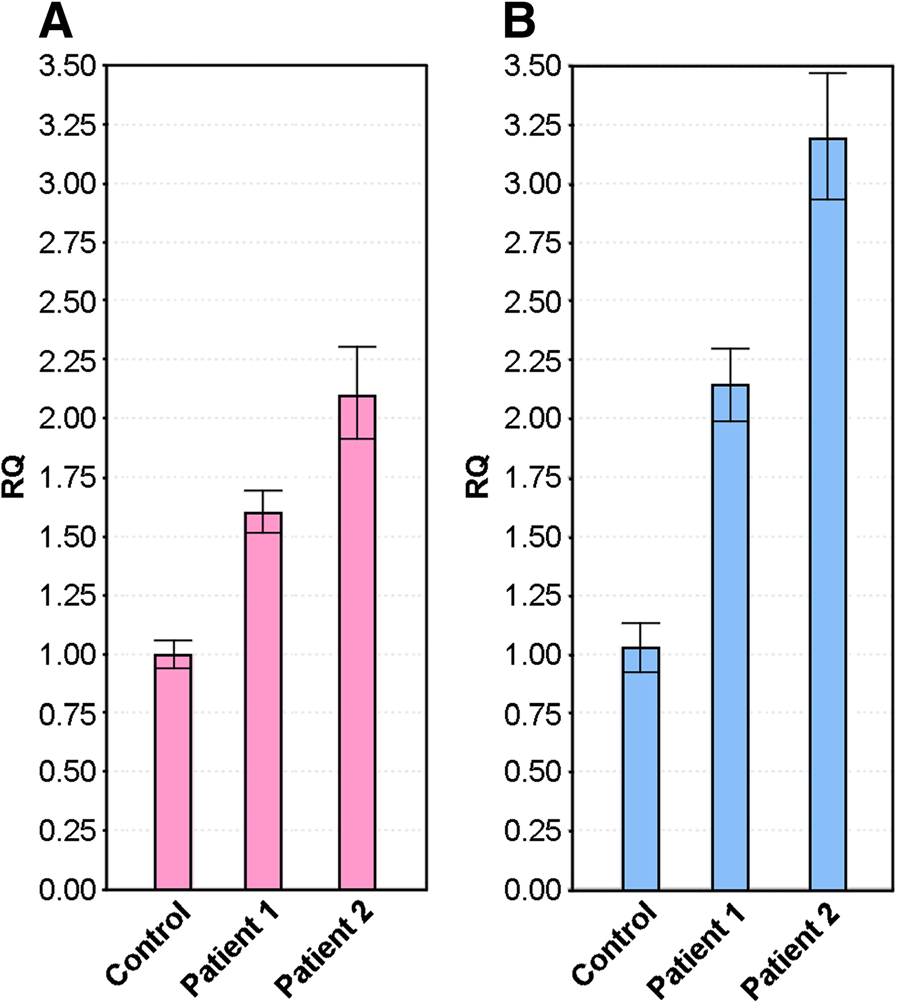
**Confirmation and quantitation of copy number using qRT-PCR. (A)** untreated genomic DNA. **(B)** bisulfite-treated DNA. (*Abbreviations:* qRT-PCR: quantitative real-time polymerase chain reaction; RQ: Relative quantification).

Gene copy number was also investigated using relative quantitative real-time polymerase chain reaction (qRT-PCR) with SYBR Green dye and *SNRPN* as the target for quantifying copy number. Analysis showed copy number of 1.604 for *SNRPN* relative to the reference gene for Patient 1 and 2.098 for Patient 2 when compared against a phenotypically normal control. This corresponds to 3 copies and 4 copies, respectively (Figure 3A).

### Methylation analysis

Sodium bisulfite treatment followed by qRT-PCR showed that both the methylated and unmethylated *SNRPN* alleles were present. Patient 1 had two methylated alleles and one unmethylated allele. For patient 2, the copy number of the methylated allele was three times the unmethylated one when compared with the control sample (Figure 3B).

### Pathway analysis

Ingenuity Pathway Analysis (IPA) using genes from the duplicated/triplicated region for Patient 2 identified developmental disorder, neurological disease and hereditary disease as the main networks affected. The broad categories of the pathways with their statistically significant *p*-values are presented in Table 2. Enrichment analysis for diseases and biological functions was performed on the constructed networks. Those with at least five molecules in the network are presented in Table 3.

**Table 2:** List of systems/processes associated with genes in the amplified region as identified by IPA

**Category**	***p*-values**	**Molecules encoded from duplicated region**
Auditory and Vestibular System Development and Function	2.20E-03	GABRA5,GABRB3
Auditory Disease	2.20E-03 - 8.41E-03	GABRA5,GABRB3
Behaviour	1.36E-03	GABRB3
Cancer	1.07E-02 - 4.42E-04	ATP10A,MKRN3,SNRPN,UBE3A
Cardiac Arteriopathy	4.33E-03	GABRA5,GABRB3,GABRG1,GABRG3
Cardiovascular Disease	4.33E-03 - 4.44E-08	GABRA5,GABRB3,GABRG1,GABRG3
Cell cycle	1.02E-02 - 4.42E-04	HERC2, UBE3A
Cell Death and Survival	3.03E-03 - 1.17E-03	GABRA5,GABRB3
Cell Morphology	2.68E-04 - 1.08E-02	OCA2, GABRA5,GABRB3,NDN
Cell-To-Cell Signalling and Interaction	1.57E-01 - 8.12E-03	NDN, UBE3A
Cellular Assembly and Organization	1.02E-02 - 7.54E-03	SNURF
Cellular Compromise	3.36E-05 - 1.72E-03	GABRA5,GABRB3
Cellular Development	8.05E-03 - 3.36E-05	NDN, UBE3A
Cellular Growth and Proliferation	8.05E-03 - 5.96E-04	NDN, UBE3A
Cellular Movement	2.68E-04 - 1.51E-04	GABRA5,GABRB3,NDN
Connective Tissue Development and Function	1.57E-01	NDN
Connective Tissue Disorders	1.40E-03 - 1.40E-08	GABRA5,GABRB3,GABRG3
Developmental Disorder	1.17E-02 - 4.69E-10	GABRB3,GABRG3,MAGEL2,MKRN3,NDN,SNRPN,UBE3A
DNA Replication, Recombination, and Repair	1.15E-02	SNRPN
Endocrine System Disorders	8.57E-03	MKRN3
Gene Expression	1.15E-02 - 6.35E-05	NDN,SNRPN,UBE3A
Hair and Skin Development and Function	4.97E-03	OCA2
Hematological Disease	5.83E-03	UBE3A
Hepatic System Development and Function	1.57E-01	NDN
Hereditary Disorder	1.17E-02 - 4.69E-10	GABRA5,GABRB3,GABRG3,MAGEL2,MKRN3,NDN,SNRPN, UBE3A
Infectious Disease	4.68E-05	GABRA5,GABRB3,GABRG3
Kidney Failure	2.45E-01	GABRB3
Liver Fibrosis	1.57E-01	NDN
Nervous System Development and Function	8.12E-03 - 1.51E-04	GABRA5,GABRB3,NDN, UBE3A
Neurological Disease	1.17E-02 - 5.59E-09	GABRA5,GABRB3,GABRG3,UBE3A
Nutritional Disease	1.82E-03 - 3.03E-06	GABRA5,GABRB3,GABRG3
Organ Morphology	5.87E-03 - 2.20E-03	GABRA5,GABRB3, UBE3A
Organismal Functions	5.87E-03	NDN
Organismal Injury and Abnormalities	2.45E-01 - 3.74E-06	GABRA5,GABRB3,GABRG3
Protein Degradation	1.33E-03	UBE3A
Protein Synthesis	1.33E-03	UBE3A
Psychological Disorders	5.57E-04 - 1.34E-06	GABRA5,GABRB3,GABRG3,NDN
Renal and Urological Disease	2.45E-01	GABRB3
Reproductive System Development	4.07E-03	UBE3A
Reproductive System Disease	8.57E-03 - 5.87E-03	MKRN3, UBE3A
Respiratory Disease	1.07E-02 - 5.97E-04	GABRA5,GABRB3,GABRG3, MKRN3, SNRPN
Skeletal and Muscular Disorders	8.12E-03 - 1.40E-08	GABRA5,GABRB3,GABRG3
Tissue Morphology	5.26E-06 - 8.57E-03	GABRA5,GABRB3,UBE3A

*Abbreviation:IPA* ingenuity pathway analysis.

**Table 3:** Diseases or functions associated with the networks constructed from genes in the amplified region

**Diseases or functions annotation**	***p*-values**	**Molecules**	**# molecules**
Prader-Willi syndrome	4.69E-10	GABRG3,MAGEL2,MKRN3,NDN,SNRPN	5
Absence seizure	5.59E-09	GABRA5,GABRB3, GABRG1*,GABRG3,UBE3A	5
Tonic-clonic seizure	2.07E-07	GABRA5,GABRB3, GABRG1*,GABRG3,UBE3A	5
Multiple congenital anomalies	4.32E-06	GABRB3,GABRG3,MAGEL2,MKRN3,NDN,PEX10*,SNRPN,UBE3A	8
Stroke	3.14E-05	GABRA5,GABRB3,GABRG1*,GABRG3,TP53*	5
Epileptic seizure	5.60E-05	GABRA5,GABRB3,GABRG1*,GABRG3,UBC*	5
Major depression	1.52E-04	GABRA5,GABRB3, GABRG1*,GABRG3,IBTK*	5
Amyotrophic lateral sclerosis	3.77E-04	GABRA5,GABRB3, GABRG1*,GABRG3,TP53*	5
Schizophrenia	4.37E-04	AP1G1*,GABRA5,GABRB3, GABRG1*,GABRG3,NDN, TP53*	7
Parkinson's disease	5.57E-04	GABRA5,GABRB3, GABRG1*,GABRG3, TP53*	5
Seizures	6.96E-04	GABRA5,GABRB3, GABRG1*,GABRG3,UBC*,UBE3A	6
Congenital anomaly of skeletal bone	1.40E-03	GABRA5,GABRB3, GABRG1*,GABRG3, TP53*	5
Weight gain	1.82E-03	AP1G1*,GABRA5,GABRB3, GABRG1*,GABRG3	5
Bleeding	9.51E-03	GABRA5,GABRB3, GABRG1*,GABRG3,TP53*	5

Notes: Table includes networks with at least five molecules.

*Molecules encoded by genes mapped to regions outside 15q11 - 13.

## Discussion

Although duplications that involve the proximal region of 15q are one of the most common rearrangements in pediatric patients with congenital disorders, only a few megabase cases have been mapped by molecular karyotyping or analyzed at the gene level. Of the duplications identified by molecular methods, the sizes ranged from about 1 million base-pairs (Mb) in a multiplex ligation-dependent probe amplification study [9] to 17.7 Mb using CGH arrays with 244 K oligonucleotide probes [10]. A few large studies have found recurrent microdeletions or duplications in the 15q11-q13 region for idiopathic epilepsies, autism, and combined schizophrenia and epilepsy [11-16]. There is another relevant region for psychiatric disorders that is more distal, with *CHRNA7* (cholinergic receptor, nicotinic subunit alpha 7) within the BP4 - BP5 region as the top candidate gene [17-19].

In a large study using published data sets, the frequency of 15q duplications is reported to be 1:494 for autism cohorts and 1:508 for clinical cohorts with intellectual disability, ASD, or multiple congenital anomalies [20]. The two patients with 15q duplication/triplication in this report were from 350 cases with developmental delay and/or multiple congenital anomalies who were prospectively recruited into our array-CGH study. The gains in the two patients had different proximal breakpoints. They appeared to share the same distal breakpoint, which is within a segmental duplication that corresponds to the BP3 region, ending after the last three exons of the *HERC2* (HECT domain and RCC1-like domain 2) which are duplicated.

No karyotype data was available for Patient 1, who has Class II duplication [21]. The duplication is likely to be an interstitial microduplication with the proximal breakpoint located within BP2. The first duplicated sequence with potential function is microRNA 4508 (gain of one copy). At least 14 genes are in the duplicated region (13 OMIM genes and 8 OMIM morbid genes). The first gene duplicated is *MKRN3* (Makorin ring finger protein 3).

The triplication in Patient 2 is in the form of an sSMC. We could not ascertain the start of the copy number gain as there was no array-CGH probe for 15p and the gain started before the recognized BP1 region and the first known gene (*CHEK2P2*), which is a pseudogene on 15q. If it was interstitial and the start was near the first array CGH probe, it would be a Class I duplication [21] with the proximal and distal breakpoints within BP1 and BP3, respectively. Alternatively, it could involve the whole p arm. Array-CGH did not detect any other genomic imbalance for this patient. The gain involves at least 22 genes out of which 18 are OMIM genes and 9 are OMIM morbid genes (Table 1). At least three genes within this region (*NIPA1*, *NIPA2*, *CYFIP1*) are implicated in the development of the central nervous system while a fourth gene, *TUBGCP5*, is a member of the cytoskeleton tubulin complex in cells and is evolutionarily conserved [22].

Although parental samples were not available for both patients, qRT-PCR analysis using primers which are specific for methylation states showed that the extra chromosomal material for both patients had methylation pattern that implied gains of maternal origin. This is consistent with the maternal origin of such duplications being more common, and with such duplications being more likely to have pathogenic consequences. Gain in copy number of maternally-derived *SNRPN* has been associated with autism [22], but Patient 2 had not presented with such features. One notable feature is that her physical dimensions were above the 90^th^ percentile. Overgrowth has been reported in patients with an increased dosage of distal 15q [23], but this patient’s copy number gain does not involve the distal 15q region.

For the two patients in this report, phenotypic severity did not correlate with the size and dosage of the distal breakpoint on 15q, and whether the duplication is interstitial or in the form of an sSMC. This lack of correlation could be due to additional factors such as presence of mosaicism in Patient 2, genetic background, epigenetic modifications, and gender. Aside from the increase in gene dosage and the parental origin of the duplicated genes, additional alterations at the epigenetic level could influence gene expression, which could lead to phenotypic variability for patients who carry duplications of the same size and dosage [21,24]. Hogart et al. provided some supporting evidence when they measured the level of 10 transcripts within the 15q11-13 region in two postmortem brains and found that the expression pattern correlated with parental gene dosage in the male patient. In the female brain, there was decreased expression of *SNRPN, NDN,* small nuclear RNAs (*snoRNAs*) and gamma-aminobutyric acide (*GABA)*_*A*_ despite an increased dosage of genes of maternal origin [25]. In the case of *SNRPN*, the decreased expression was consistent with the finding of increased methylation found at the imprinting control region.

Mosaicism is common in 15q duplications that involve supernumerary derivative chromosome 15. Such duplications tend to take the form of pseudodicentric derivative chromosomes rather than intrachromosomal. The pseudodicentric marker chromosome, psu dic(15;15), is usually formed by a homologous recombination between two chromosomes 15 [26]. The smaller marker chromosome in Patient 2 is likely to be the result of a break in the psu dic(15;15), before the inactivation of one centromere which occurred during the anaphase stage of mitosis. The break would also have resulted in the loss of one of the two duplicated regions in some cells, giving rise to the mosaicism observed. As karyotype analysis was only done for cells from peripheral blood culture, the ratio of the two marker chromosomes in other tissues is not known. At the time of the blood sampling for genetic investigation, the patient was only 9 months old. Due to the young age when the chromosomal studies were done and the lack of data on the mosaicism level in other tissues, it is difficult to predict the course of disease manifestation and make genotype-phenotype correlation [27].

Genetic analysis determined that the genes within the regions that were duplicated in one or both of the patients are significantly associated with identified functional networks. The top three functional networks based on levels of statistical significance are developmental disorder, hereditary disorder, and neurological disease. The important genes that are involved in developmental and neurological disorders are *GABRA5, GABRA3, GABRG3, MAGEL2, MKRN3, NDN*, *SNRPN* and *UBE3A*. Three of the genes encode subunits of the GABA receptors, a family of ligand-gated chloride channels which mediate the major inhibitory neurotransmitter GABA in the brain. They have been found to be highly expressed in the cerebral cortex of postmortem brain samples [28]. One study found that duplications that involve this GABA gene cluster are highly enriched in an autism cohort, while another study found no difference from controls [16,29]. The imprinted gene *UBE3A* functions as a transcriptional co-activator and also as a ligase in the ubiquitin proteasome pathway. *SNRPN* also has dual functions. It is involved in RNA processing and is also spliced into several regulatory RNAs. The remaining three genes are causative of Prader-Willi syndrome if a deletion or mutation is of paternal origin. All are intronless, transcribed only from paternal alleles and are involved in growth regulation or transcription. In addition, there are a number of C/D box *snoRNAs* which occur in multiple tandem copies [30]. These include the SNORD 115 (HBII-52), SNORD 116 (HBII-85) and SNORD 109A (HBII-438A) clusters. They are involved in directing alternative splicing or site-specific methylation of substrate RNAs [31,32]. However, it is unclear whether they are important in cases of maternal duplications (such as the two cases in this report) as they were reported to be expressed from the paternal chromosome only [33].

## Conclusions

We report two new cases of trisomy and mosaic tetrasomy 15q11-q13 of probable maternal origin from the methylation pattern. The copy number gain of genes in the region could explain the patients’ phenotypic presentations. Pathway analysis identified multiple networks of candidate gene interactions. Reports of additional cases that have overlapping amplifications with different breakpoints would be helpful toward delineating the spectrum of phenotypic features and long-term follow-up for carriers of such amplifications, such as the development of late-onset Lennox-Gastaut syndrome [34] and sudden unexplained deaths [5].

### Consent

Written informed consent was obtained from the parents for the laboratory investigations and publication of the case report.

## Methods

### Karyotype analysis

Chromosome analyses were performed on GTG banded metaphases obtained from cultures of phytohaemagglutinin-stimulated lymphocytes using standard methods. High-resolution chromosomes were obtained by Methotrexate cell synchronization [35].

### Fluorescence in situ hybridization

Targeted cytogenetic analysis was performed on metaphase spreads using bacterial artificial chromosome probes obtained from The Hospital for Sick Children (Toronto, Canada). Slides were counterstained with 4’,6-diamidino-2-phenylindole in Vectashield mounting medium (Vector Laboratories, Inc, USA) and analyzed using a fluorescence microscope Olympus BX51 equipped with a CCD Progressive Scan Video Camera (Japan Analytical Industry, Co. Ltd., Japan). Image analysis was carried out with Cytovision software (version 3.93.2) (Applied Imaging Corp, USA).

### Array-based copy number analysis

Genomic DNA was extracted from peripheral blood using the Puregene DNA Isolation Kit (Qiagen GmbH, Hilden, Germany). Human CGH array consisting of 400 K 60-mer oligonucletode probes from Agilent (Agilent Technologies Inc., Santa Clara, CA, USA)—and the reference used was human genomic DNA from Promega matched to the gender of the patient (Promega Corp., Madison, WI, USA). Test DNA was labeled with Cy5-dUTP and reference DNA was labeled with Cy3-dUTP (Sigma-Aldrich, St. Louis, MO, USA) according to Agilent’s protocol for enzymatic labeling (Version 6.3). The efficiency of the labeling was measured using a Nanodrop Spectrophotometer. The labeled reference and test DNA samples were hybridized to the array at 65°C in an Agilent Hybridization Oven for 40 hours, with the rotator set at 20 rotations per minute. The array was then processed according to the manufacturer’s instructions and scanned with an Agilent G2505C Microarray scanner at 5 micron resolution. Data were extracted from the scanned image using Agilent Feature Extraction (Version 10.7.31) and were analyzed for copy number change using Agilent Genomic Workbench Lite (Edition 6.0.130.24). Genomic coordinates are based on genome build 36/hg18.

### qRT-PCR analysis

Gene copy number was also investigated by relative qRT-PCR with SYBR Green dye and *SNRPN* as the target for quantifying copy number. Primers were designed using Primer Express (Version 3.0), and the experiment was carried out in triplicate. Genomic DNA from the two patients and a control participant was amplified in the same experiment using *ZNF80* as the internal reference [36]. Amplification was done using the Applied Biosystems StepOnePlus real time PCR system (Applied Biosystems Incorporated, Foster City, CA, USA). Results were analyzed using Applied Biosystems StepOne software (version 2.1).

### Methylation analysis

Methylation status was investigated by treating the DNA with sodium bisulfite using the EpiTect Bisulfite Kit (Qiagen GmbH, Hilden, Germany), followed by qRT-PCR with separate primers targeting methylated and unmethylated *SNRPN* according to Kubota et al. [37].

### Pathway analysis

The coordinates of the minimum deleted region for Patient 2 was searched against the Human reference genome (hg18) for known genes in the region. The resulting list of genes was imported into IPA software (Ingenuity Systems, Inc. Redwood City, CA, USA) using the Entrez ID mapped to the Ingenuity Pathway Knowledge Base identifier. The reference set used was Ingenuity Knowledge Base (Genes only); relationship to include was both direct and indirect. The analysis included endogenous chemicals, and the filter summary was set to consider only relationships in which confidence = experimentally observed. The statistical significance for the enrichment of genes of interest in each pathway was evaluated using a Fisher Exact test under the Core Analysis function of IPA.

## Abbreviations

BP: Breakpoint; bp: Basepairs; CGH: Comparative genomic hybridization; FISH: Fluorescent in situ hybridization; GABA: Gamma aminobutyric acid; IPA: Ingenuity pathway analysis; Mb: Million base-pairs; OMIM: Online Mendelian inheritance in man; qRT-PCR: Quantitative real-time polymerase chain reaction; sSMC: Supernumerary marker chromosome; *SNRPN*: Small nuclear ribonucleoprotein polypeptide N.

## Competing interests

ZHL is an employee of Genomax Technologies Pte Ltd, distributor of Agilent products in Singapore. There is no competing interest for the other authors.

## Authors’ contributions

ECT conceived the project, obtained the funding, and participated in writing the first draft of the manuscript and in performing the array-CGH analysis; EST did the clinical assessments and participated in writing the first draft of the manuscript; ECPL performed the array-CGH and qRT-PCR experiments; MHY oversaw the karyotyping and performed the FISH analysis; ZHL did the pathway analysis; MSB participated in performing the array-CGH analysis. All authors read and approved the manuscript.

## Authors’ information

EST: Senior Consultant, Genetics Service, KK Women’s and Children’s Hospital, Singapore. MHY: Principal Scientific Officer, Cytogenetics Laboratory, KK Women’s and Children’s Hospital. ECPL: Senior Medical Technologist, KK Women’s and Children’s Hospital. ZHL: Application Manager (Bioinformatics), Genomax Technologies, Singapore. MSYB: Principal Scientist, KK Research Centre, KK Women’s and Children’s Hospital. ECT: Principal Scientist, KK Research Centre, KK Women’s and Children’s Hospital and Adjunct Associate Professor, Office of Clinical Sciences, Duke-NUS Graduate Medical School Singapore, Singapore.
